# Fibronectin as a Prognostic Indicator in Portal Hypertension

**DOI:** 10.1155/1992/45283

**Published:** 1992

**Authors:** Ruth F. Mckee, D. P. Maharaj, O. J. Garden, D. C. Carter

**Affiliations:** 1 University Department of Surgery and Department of Haematology Royal Infirmary Glasgow UK; 2 University Department of Surgery Royal Informary Glasgow G4 0SF UK; 3 Ward 50 Aberdeen Royal Infirmary Foresterhill Aberdeen AB9 2ZB Scotland

## Abstract

Plasma fibronectin levels were measured in 33 patients with portal hypertension and c6mpared with modified Child’s grading and a previously described prognostic index. Outcome at one year from blood sampling was recorded. Mean plasma fibronectin level was 304.1 mg/ml (sem 24.3) and significantly lower levels were found in patients who had had a variceal bleed within the previous seven days. Plasma fibronectin levels tended to be lower in patients with poor liver function as assessed by modified Child’s grading but this did not achieve statistical significance. Plasma fibronectin alone was not an accurate predictor of one year survival in these patients but only one of seven patients who had a plasma fibronectin level below 300mg/l in association with a poor prognostic index survived for one year.


*HPB Surgery*, 1992, Vol. 5, pp. 229–234
Reprints available directly from the publisher
Photocopying permitted by license only

© 1992 Harwood Academic Publishers GmbH
Printed in the United Kingdom

### FIBRONECTIN AS A PROGNOSTIC INDICATOR IN PORTAL HYPERTENSION

RUTH F.MCKEE, D.P. MAHARAJ, O.J. GARDEN AND D.C.CARTER
*University Department of Surgery and Department of Haematology, Royal Infirmary, Glasgow, UK*

*(Received 20 December 1990)*

Plasma fibronectin levels were measured in 33 patients with portal hypertension and cômpared with modified Child's grading and a previously described prognostic index. Outcome at one year from blood sampling was recorded.

Mean plasma fibronectin level was 304.1 mg/ml (sem 24.3) and significantly lower levels were found in patients who had had a variceal bleed within the previous seven days. Plasma fibronectin levels tended to be lower in patients with poor liver function as assessed by modified Child's grading but this did not achieve statistical significance.

Plasma fibronectin alone was not an accurate predictor of one year survival in these patients but only one of seven patients who had a plasma fibronectin level below 300mg/l in association with a poor prognostic index survived for one year.

KEY WORDS: Fibronectin, portal hypertension

### INTRODUCTION

Fibronectin is a glycoprotein which is produced by vascular endothelial cells, fibroblasts and hepatocytes[1] and is thought to function as an opsonin during phagocytosis. Low fibronectin levels have been associated with poor survival in patients with sepsis and trauma[2].

Abnormal levels of plasma fibronectin have been found in patients with liver disease. Whereas increased levels have been found in uncomplicated acute hepatitis[3,4], low levels were noted in patients with fulminant hepatic failure[5]. Cirrhotic patients with portal hypertension have been reported to have low levels of plasma fibronectin[6] and it has been suggested that plasma fibronectin level correlates with survival in these patients[7].

The aim of this study was to assess plasma fibronectin levels in a variety of portal hypertensive patients, to determine whether plasma fibronectin was related to the severity of liver disease as assessed by Pugh's modification of Child's grading[8] and a previously reported prognostic index[9], and to relate plasma fibronectin level to prognosis.

Address correspondence to: Dr Ruth F. McKee, University Department of Surgery, Royal Informary, Glasgow G4 0SF, UK current address Ward 50, Aberdeen Royal Infirmary, Foresterhill, Aberdeen AB9 2ZB

### PATIENTS AND METHODS

Thirty-three patients with portal hypertension and a history of bleeding oesophageal varices were studied. Details of the patients' age, sex, liver disease and modified Child's grading are shown in Table 1. All patients had endoscopically proven oesophageal varices and the presence of liver disease was confirmed on the basis of history, clinical findings and by liver biopsy, when the coagulation screen allowed this, or isotope liver scan. Only one patient had undergone portacaval shunting previously and this shunt was known to be thrombosed at the time of study. Splenectomy had been performed in two other patients. Plasma samples were obtained from 33 patients during the patient's first admission to hospital during the study period, either following a variceal bleed[13] or for elective sclerotherapy[20]. In thirteen patients repeat plasma fibronectin samples were obtained during a subsequent admission at least four weeks after the first sample had been taken. Blood samples were spun down immediately and stored at – 20SoT, 4C until analysis could be performed. Immunological levels of fibronectin were then measured using a turbidometric method (Boehringer-Mannheim Kit No 401218).

At the time of removal of each plasma sample details of the patient's current clinical findings, full blood count, coagulation screen, urea and electrolytes, glucose and liver function tests were recorded. Liver function was assessed by Pugh's modification of Child's grading[8] and by the use of a prognostic index[9]. To obtain this index the probability of 1 year survival($p$) was calculated using the equation

$$1n(p/(1-p)) = 7.8 - 0.76ENC - 2.7PTR - 0.05CR$$

**Table 1** Patient details divided with regard to whether they had experienced a variceal bleed in the seven days prior to initial blood sampling or not

| | *Age* | *Sex ratio* | *Aetiology* | *Modified Child's Grading* |
|---|---|---|---|---|
| Acute | 60.3 | 11:2 | 10 Alcohol cirrhosis<br>1 Primary biliar cirrhosis<br>1 Chronic active heptatitis<br>1 Drug induced cirrhosis | 2-A: 3-B: 8-C |
| Stable | 57.3 | 13:7 | 12 Alcoholic cirrhosis<br>4 Primary biliary cirrhosis<br>1 Chronic active hepatitis<br>2 Cryptogenic cirrhosis<br>1 Portal vein thrombosis | 8-A: 8-B: 4-C |
| Total | 58.5 | 24:9 | 22 Alcoholic cirrhosis<br>5 Primary biliary cirrhosis<br>2 Chronic active hepatitis<br>2 Cryptogenic cirrhosis<br>1 Drug induced cirrhosis<br>1 Portal vein thrombosis | 10-A: 11-B: 12-C |

where the actual values of prothrombin ratio(PTR) and creatinine(CR) are entered and encephalopathy(ENC) is coded as –1 when absent and +1 when present. The date of the most recent variceal bleed was noted, along with any treatment necessary, in particular recent blood or blood product transfusions and injection sclerotherapy. A recent acute bleed was defined as haematemesis or melaena in the previous seven days causing either shock (pulse rate > 100; systolic blood pressure < 100), a fall in haemoglobin to less than 10g/dl or a drop in haemoglobin level of 3g within 48 hours. Survival was assessed for each patient from the first admission to hospital while in the study and for at least one year after this admission.

Unpaired *t*-tests were used for comparison of plasma fibronectin levels in different groups of patients. Paired *t*-tests were used to compare two successive fibronectin and prognostic index measurements in 13 patients.

### RESULTS

The mean initial plasma fibronectin for all patients was 304.1mg/l.(sem 24.3) Only two patients had plasma fibronectin levels below the normal range for our laboratory (140–490mg/l). The mean fibronectin level in the group of patients within seven days of a variceal bleed was 236.8mg/l (sem 29.1, $n=13$) compared to 347.8mg/l (sem 32.2, $n=20$) in the group of stable patients. The difference between these two groups was significant ($t=2.39$, $p<0.05$). A low plasma fibronectin level was not associated with clinical findings of ascites or splenomegaly. Plasma fibronectin level varied with liver function, the mean fibronectin level in modified Child's A and B patients being 327.5mg/l (sem 31.9, $n=21$) while in modified Child's C patients the mean fibronectin level was 263mg/l (sem 35, $n=12$). However, this difference was not statistically significant. Plasma fibronectin level did not correlate with prognostic index (Figure 1; $r=-0.1$).

Three patients died during the index admission for this study, all following a variceal bleed. A further eight patients died during the next year, twenty-two patients survived. Two of these patients died of causes unrelated to their liver disease. The level of plasma fibronectin alone was not an accurate predictor of survival over the following year (Figure 2), although the mean plasma fibronectin in one year survivors (337.5mg/l, sem 32.3) was significantly higher than in those patients who died (237.2mg/l, sem 24.6: $t=2.47$, $p<0.01$). However plasma fibronectin level and prognostic index together gave good prediction of 1 year survival (Figure 1). Of seven patients who had a plasma fibronectin level of less than 300mg/l along with a prognostic index of less than 0.5, six died over the next twelve months.

When the 13 patients with repeat plasma fibronectin samples were studied, in nine patients who were stable on both admissions there was no significant difference between the value of prognostic index or plasma fibronectin (mean first fibronectin value = 337.5mg/l sem 67.5; mean second fibronectin = 303.3mg/l sem 39.0; $t=0.56$; $p<0.5$).

### DISCUSSION

Few portal hypertensive patients in the present study had plasma fibronectin levels

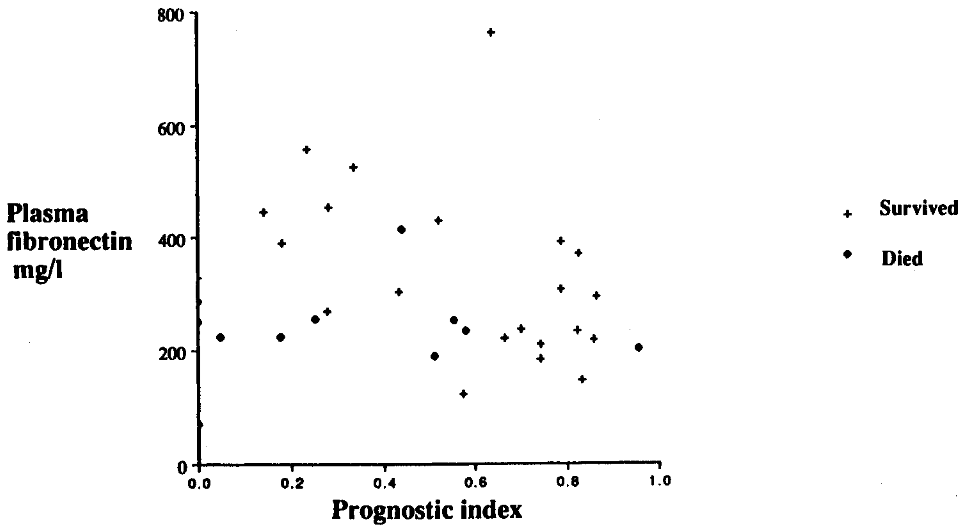

**Figure 1** Plasma fibronectin level (mg/l) and prognostic index (9) in 33 patients with portal hypertension. Patients who survived to one year after blood sampling shown as crosses (+), patients who died within one year shown as closed circles (•).

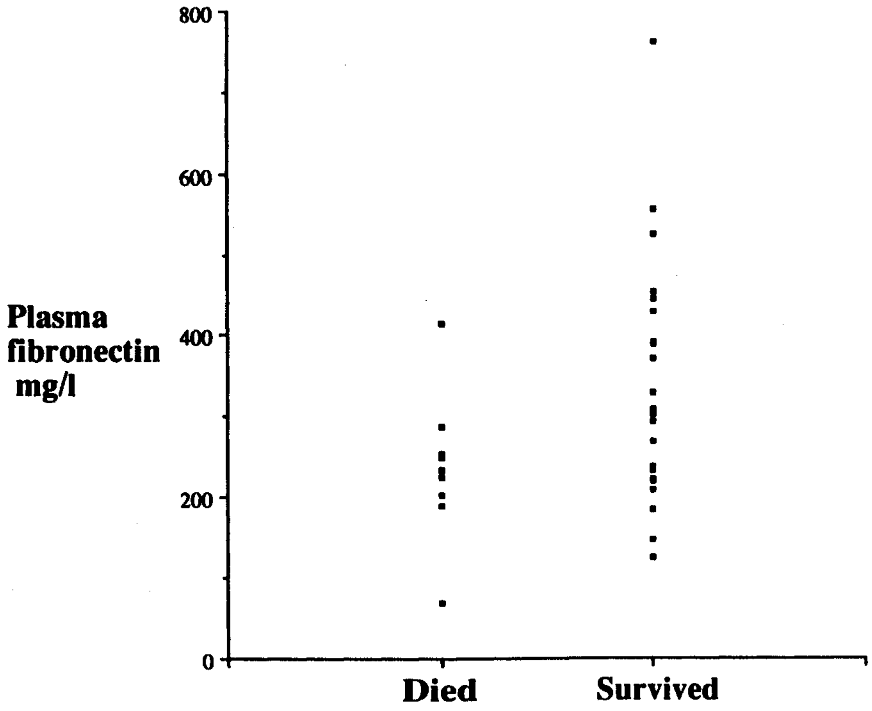

**Figure 2** Plasma fibronectin level compared to one year survival in 33 patients with portal hypertension.

below the normal range. Although Foster and colleagues[6] found reduced plasma fibronectin levels in portal hypertension Gluud's group[4] could not confirm this finding and Matsuda and colleagues[3] could only show a reduction in plasma fibronectin in patients with decompensated cirrhosis. Although some of our patients had severe hepatic decompensation with ascites, encephalopathy and grossly abnormal liver function, the majority had apparently well preserved liver function, as assessed by modified Child's grading. There was a tendency towards lower plasma fibronectin values in grade C patients although this was not significantly different from grade A and B patients. Plasma fibronectin has also been shown to be raised in acute hepatitis [3,4] and these variations in liver function may account for the wide range of plasma fibronectin levels in our patients.

In the present study, plasma fibronectin levels have been found to be lower following recent variceal haemorrhage. Blood in the gastrointestinal tract might lead to changes in the bacterial population and the associated hypotension might lead to episodes of endotoxaemia during variceal haemorrhage. In a recent preliminary study we have demonstrated endotoxaemia in several patients during the 48 hours after a variceal bleed. The depletion in plasma fibronectin levels may therefore result from its use in increased reticulo-endothelial system activity.

We have been unable to show that plasma fibronectin alone is an accurate indicator of survival in patients with portal hypertension. In these patients many factors are involved in determining prognosis and it seems unlikely that a single variable can predict outcome accurately in a heterogenous group of portal hypertensive patients. It is difficult to separate the effects of a variceal bleed, poor liver function and any other variable which might affect outcome as these variables are interrelated.

Nonetheless the combination of low plasma fibronectin and a low prognostic index was associated with a very poor prognosis (Figure 1). In this study, stable patients have been included as well as patients who had had a recent variceal bleed and this may explain the reduction in the accuracy of predicting prognosis from 81% to 68% when the prognostic index is used alone. However when prognostic index and plasma fibronectin level are taken together only one of the seven patients with a prognostic index of less than 0.5 and plasma fibronectin level of less than 300mg/l survived to one year. Since fibronectin acts as an opsonin in the reticuloendothelial system it is not surprising that low levels of this glycoprotein in association with indicators of poor hepatic and renal function are associated with a poor prognosis in portal hypertension.

In conclusion, we have shown that in a heterogenous group of patients with portal hypertension there is a wide range of plasma fibronectin levels, and this may be due to differeing degrees of hepatic decompensation and hepatitis. A recent variceal bleed is associated with lower plasma fibronectin levels.

### *References*

1. Owens M.R. and Cimino, C.D. (1982) Synthesis of fibronectin by the isolated perfused rat liver. *Blood*, <bs59, 1305–1309
2. Saba,T.M. (1986) Plasma fibronectin. *Br. J. Hosp. Med.*, **37**, 364–366
3. Matsuda, M., Yamanaka, T. and Matsuda A. (1982) A Distribution of fibronectin in plasma and liver in liver diseases. *Clinica Chimica Acta*, **118**, 191–199
4. Gluud, C., Dejgaard, A. and Clemmensen, I. (1983) Plasma fibronectin concentrations in patients with liver diseases. *Scand. J. Clin. Lab. Invest.*, **43**, 533–537

5. Gonzalez-Calvin, J., Scully, M.F., Sanger, Y., Fok, J., Kakkar, V.V., Hughes, R.D., Gimson, A.E.S. and Williams,R. (1982) Fibronectin in fulminant hepatic failure. *Br. Med. J.*, **2**, 1231–1232
6. Foster, P.N., Bowen, M., Howdle, P.D. and Losowsky, M.S. (1983) Low fibronectin in portal hypertension. *Digestion*, **28**, 122–124
7. Naveau, S., Poynard, T., Abella, A., Pignon, J-P., Poitrine, A., Agostini, H., Zourabichvili, O. and Chaput, J-C. (1985) Prognostic value of serum fibronectin concentration in alcoholic cirrhotic patients. *Hepatology,* **5**, 819–823
8. Pugh, R.N.H., Murray-Lyon, I.M., Dawson, J.L., Pietroni, M.C. and Williams R. (1973) Transection of the oesophagus for bleeding varices. *Br. J. Surg.*, **60**, 646–649
9. Garden, O.J. Gilmour, W.H. and Carter, D.C. (1985) Factors affecting one-year survival following acute variceal haemorrhage. *J. Roy. Coll.Surg.,Edin.*, **30**, 277–282

(*Accepted by S. Bengmark 25 January 1991*)

